# ﻿Two new species of the genus *Kingdonella* Uvarov, 1933 (Orthoptera, Acridoidea) and the first reported male of *Kingdonellaqinghaiensis* Zheng, 1990

**DOI:** 10.3897/zookeys.1205.127999

**Published:** 2024-06-27

**Authors:** Jianyu Chen, Yulong Zhang, Xinjiang Li

**Affiliations:** 1 The Key Laboratory of Zoological Systematics and Application, School of Life Sciences, Institute of Life Sciences and Green Development, Hebei University, Baoding 071002, China Hebei University Baoding China

**Keywords:** Grasshopper, identification key, Melanoplinae, taxonomy

## Abstract

Two new species of the genus *Kingdonella* and the first report of a male *K.qinghaiensis* Zheng, 1990 are presented. The new species *K.gandensis***sp. nov.** has similar morphological features to *K.wardi* Uvarov, 1933, but it differs from the latter in having 1) the hind tibia black; 2) the epiproct, in males, with a median groove in the basal 1/2 and in the apical 1/4; 3) the denticles of the male epiproct black; 4) the outside of the hind femur reddish-brown on the basal 1/4 and black on the apical 3/4; and 5) the ventral face of the hind femur black on the outer side. The second new species, *K.biruensis***sp. nov.**, is morphologically close to *K.pienbaensis* Zheng, 1980 but differs from the latter in having 1) the length of the middle segment (12th segment) of antennae 1.2 times longer than its width; 2) the subgenital plate sharp-cornered in males; 3) the ovipositor smooth; 4) the upper half of hind femur outside surface with two black spots; and 5) the ventral face of the hind femur black on its outer side, red on the basal 2/3, and black on the apical 1/3 of its inner side. Finally, we provide a key to all known species of *Kingdonella*.

## ﻿Introduction

The genus *Kingdonella* was established by Uvarov in 1933 with *K.wardi* Uvarov, 1933 as the type species (Uvarov 1933). *Kingdonella* is endemic to the Qinghai-Xizang Plateau. All species in this genus lack tegmina, wings, and a tympanum, which are representative evolutionary adaptations to the plateau environment at altitudes of 3000–5000 m.

*Kingdonella* was extensively studied in the 20th century, and 17 species in the genus have been described (Uvarov 1933, 1935, 1939; Mishchenko 1952; Zheng 1980, 1990; Huang 1981; Yin 1984; Li and Xia 2006; Li and Yin 2009). Due to the absence of tegmina and wings, these species have restricted capacity for dispersion and migration across the plateau, leading to narrow distribution ranges. Upon meticulously examining our collected specimens, we identified two new species of *Kingdonella* in the locales of Gande, Qinghai and Biru, Xizang. Additionally, we encountered the male of *K.qinghaiensis* within the confines of Zhiduo, Qinghai, which is the precise location where the holotype was collected. Finally, a key to the genus *Kingdonella* is revised and presented.

## ﻿Materials and methods

All samples were collected from Qinghai and Xizang, China. After collection, samples were dried for morphological and color description. Potassium cyanide was used during the drying process to preserve the natural colors. The type specimens were deposited in the School of Life Sciences, Hebei University, Baoding, China. The specimens were photographed using a Fujifilm XH2 camera with an XF 30 mm macro lens.

In the morphological analyses, measurements were made using the MATO software (Liu et al. 2023) for the following body characters:

Body length – dorsally from the fastigium vertex to the distal end of the abdomen.

Pronotum length – dorsally, along the median carina.

Hind femur length – laterally, maximum possible measurement of the hind femur.

## ﻿Results

### ﻿Taxonomy

**Acridoidea MacLeay, 1821**

**Acrididae MacLeay, 1821**

**Melanoplinae Scudder, 1897**

**Podismini Jacobson, 1905**

#### ﻿ Kingdonella gandensis sp. nov.

Taxon classificationAnimaliaOrthopteraAcrididae

B59A791A-884B-5524-9994-7E36F6F061B8

https://zoobank.org/75C1D661-EA93-41AE-87D6-C61B988C6416

Fig. 1

##### Type material.

***Holotype***: China • 1♂; Qinghai, Gande; 33.96°N, 99.93°E; 22.VIII.2008; coll. Xinjiang Li, Jiantao Xiao, Yongchao Zhi; catalogue number 080822015.

***Paratypes***: China 8♂ 11♀, same data as the holotype; catalogue numbers 080822016–080822035.

##### Etymology.

The species is named after Gande, the type locality.

##### Diagnosis.

The new species resembles *K.wardi* in having a small and obtuse furcula in the male epiproct and long cerci that nearly reach the tip of the epiproct, but it can be distinguished by the characters presented in Table 1.

**Table 1.** Comparison of *Kingdonellagandensis* sp. nov. and *K.wardi* Uvarov, 1933.

Characters	*K.gandensis* sp. nov.	*K.wardi*
Colors of hind tibia	Black	Red
Epiproct in male	With median groove on basal 1/2 and apical 1/4	With median groove on base
Denticles of male epiproct	Black	Red
Outer side of hind femur	Basal 1/4 reddish-brown and apical 3/4 black	Basal 2/3 red and apical 1/3 black
Outer side of ventral face of hind femur	Black	Red

##### Description.

**Figure 1.**
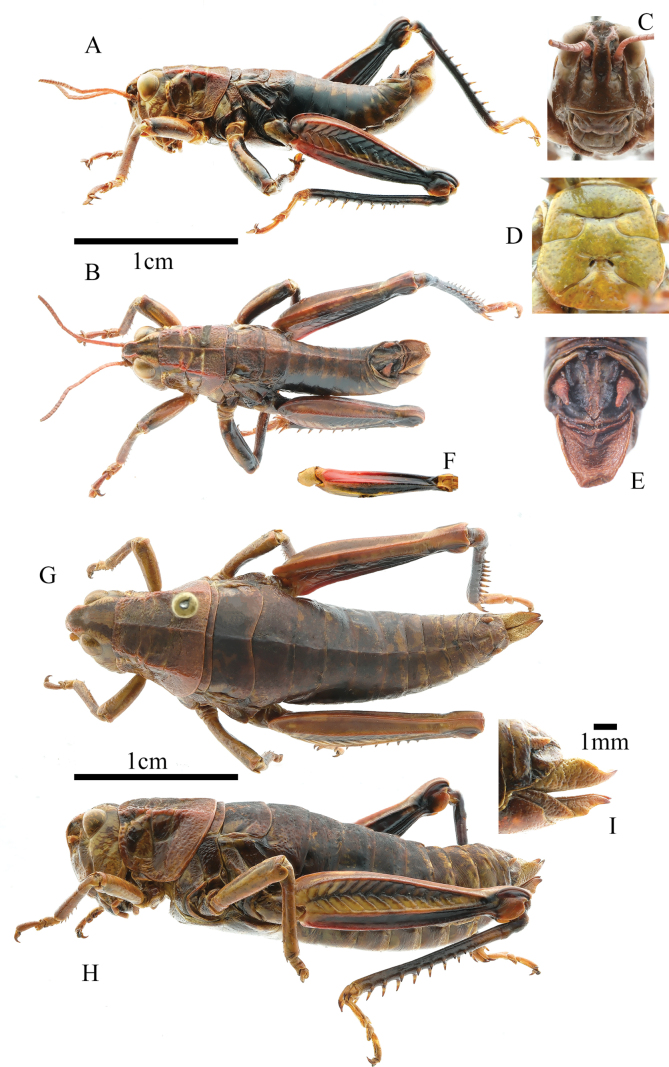
*Kingdonellagandensis* sp. nov. male **A** lateral view of holotype **B** dorsal view of holotype **C** head of holotype **D** sternal plate of male holotype, ventral view **E** terminal of abdomen, dorsal view **F** Hind femur, ventral view **G** dorsal view of female paratype **H** lateral view of paratype **I** ovipositor, lateral view.

**Male**: body medium-sized (Fig. 1A, B). Head shorter than pronotum, and frons slightly oblique in profile. Frontal costa distinct and concave on the level of median ocellus (Fig. 1C). Vertical diameter of eyes 1.1 times that of transverse and subocular furrows. Antennae filiform, with 24 segments, slightly longer than head and pronotum combined; length of a middle segment (12th segment) 2.0 times its width. Pronotum rough, nearly straight on anterior margin, and slightly depressed on median posterior margin. Median carina distinct, slightly cut by last transverse sulci; lateral carinae conspicuous, gradually expanding outward; prozona 2.0 times that of metazona in length. Prosternal process conical, bluntly round at apex. Width of metasternal lobes 1.5 times the length; minimum width of mesosternal interspace 1.6 times the length. Metasternal lobes separated and distinct (Fig. 1D). Tegmina and wings absent. Upper carina of hind femur smooth. Hind femur 4.0 times longer than wide. Outer side of upper basal lobe of hind femur distinctly longer than the lower one. Hind tibia with nine spines on inner and eight on outer side; external apical spine absent. Arolium between claws large, widely rounded, nearly reaching the apex of claws. Posterior margin of 10th abdominal tergite with distinct furcula (Fig. 1E). Tympanal organ absent. Epiproct with denticles on the middle of both sides, with a longitudinal groove in basal 1/2 and in apical 1/4, apically sharp-cornered. Cerci long, conical, and nearly reaching tip of epiproct. Subgenital plate long, sharp-cornered, and protruding backwards. In phallic complex, apical valves of penis slightly longer than cingulum valves; apodemes longer than basal valves of penis, with slightly enlarged and flaky ends; lateral view of basal valves of penis reveals a reniform shape (Fig. 4A–C). Bridge of the epiproct straight, wide, and short; ancorae angular, curving inward, with non-sharp tips; anterior projections semicircular, lateral plates straight, and posterior projections conical; lophi large and flaky, extending towards the medial and lower sides, protruding noticeably in the overall plane, with evident tumor protrusions along edge (Fig. 4D–F). **Female**: body robust, larger than male (Fig. 1G, H). Eyes small, with vertical diameter equal to transverse diameter, and 0.8 times longer than subocular furrows. Pronotum wider, trapezoidal, last transverse sulci not cutting median carina. Minimum mesosternal interspace width 2.8 times length. Ovipositor margin smooth, with a hook-like apex (Fig. 1I). Other characteristics resemble the male.

##### Coloration.

Body reddish-brown. Eyes yellowish-brown. Antennae light red. Apex and lateral margins of fastigium red. Pronotum lateral carinae red. Upper side of femur reddish-brown. Inner side of hind femur red at the base and the remaining black. Outer side of hind femur reddish-brown (yellow in females) on basal 1/4 and black on apical 3/4; Ventral face of hind femur red on basal 1/2 and black on apical 1/2 (Fig. 1F). Upper lateral genicular lobe black, other brown. Lower lateral genicular lobe red. Hind tibia black and tarsus brown. Denticles of male epiproct black.

##### Measurements.

Shown in Table 2.

**Table 2.** Measurement of *Kingdonellagandensis* sp. nov.

Number of specimens	Male			Female		
	Body length	Pronotum length	Hind femur length	Body length	Pronotum length	Hind femur length
1	17.53	4.18	10.56	29.00	5.27	12.48
2	18.04	4.28	10.64	25.29	5.82	12.98
3	18.88	4.20	10.02	25.51	6.24	12.27
4	18.15	4.44	10.55	26.73	5.50	12.91
5	18.90	4.22	10.22	22.43	5.74	12.59
6	18.39	4.20	10.33	27.91	5.40	12.93
7	19.41	4.53	10.95	22.87	5.09	12.73
8	17.71	4.42	9.88	25.85	5.51	13.06
9	18.54	4.17	10.94	27.42	5.54	13.24
10	–	–	–	23.64	5.38	12.55
11	–	–	–	28.90	5.46	14.16
Min	17.53	4.17	9.88	22.43	5.09	12.27
Max	19.41	4.53	10.95	29.00	6.24	13.24
Median	18.39	4.22	10.55	25.85	5.51	12.91

##### Distribution.

Gande, Qinghai Province, China.

#### ﻿ Kingdonella biruensis sp. nov.

Taxon classificationAnimaliaOrthopteraAcrididae

D4A2BF75-F7B3-58C3-B06C-20DF72EE5874

https://zoobank.org/0C7A3718-969C-4541-9389-F452156A1D1D

Fig. 2

##### Type material.

***Holotype***: China • 1♂; Xizang, Biru; 93.91°N, 31.27°E, 8.VIII.2009, coll. Daochuan Zhang and Yulong Zhang; Catalogue number: #090808001.

***Paratypes***: China 8♂ 8♀, same data as the holotype; Catalogue number: #090808002— #090808017.

##### Etymology.

The species is named after Biru, the type locality.

##### Diagnosis.

The new species *Kingdonellabiruensis* sp. nov. resembles *K.pienbaensis* in having the male epiproct with large denticles, but it can be distinguished by the characters presented in Table 3.

**Table 3.** Comparison of *Kingdonellabiruensis* sp. nov. and *K.pienbaensis* Zheng, 1980.

Characters	*Kingdonellabiruensis* sp. nov.	*K.pienbaensis*
Length of 12th segment in male antennae	1.2 times the width	1.6 times the width
Subgenital plate in male	Sharp-cornered	Bluntly rounded
Ovipositor	Smooth	Blunt spines in the upper valves
Upper outside of hind femur color	Yellow with two black spots	Dark
Ventral face of hind femur	Black on outer side, red on basal 2/3, and black on apical 1/3 of inner sides	Red

##### Description.

**Figure 2.**
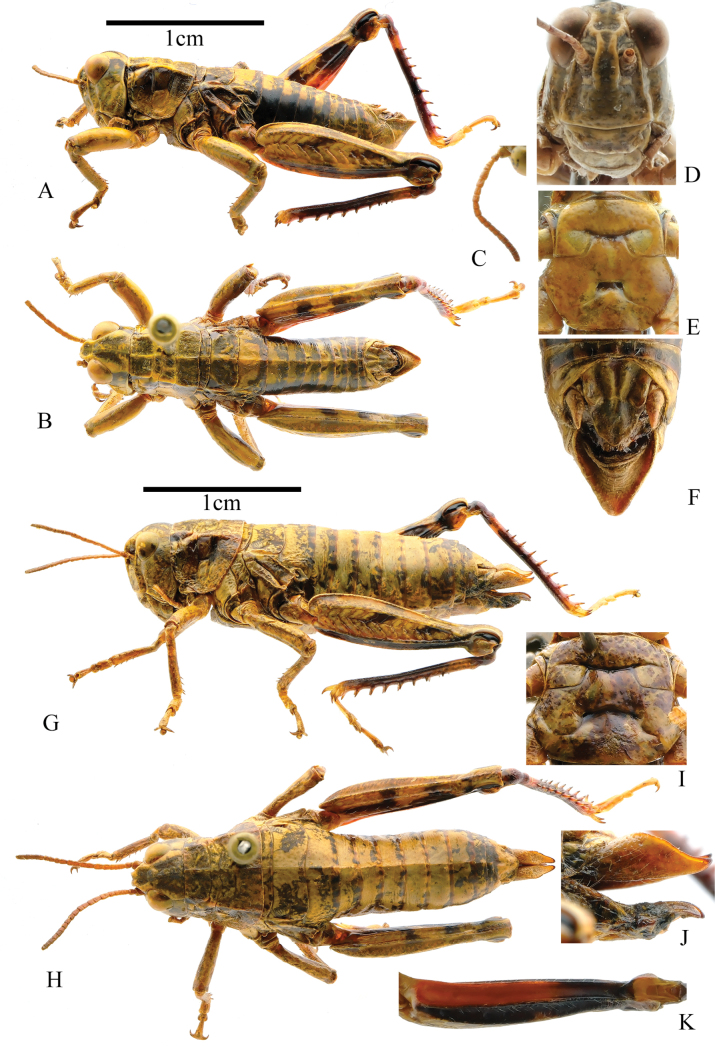
*Kingdonellabaigasis* sp. nov. **A** dorsal view of male holotype **B** lateral view of male holotype **C** antennae, dorsal view **D** head of holotype **E** sternal plate of male holotype, ventral view **F** terminal of abdomen, dorsal view **G** lateral view of female paratype **H** dorsal view of female paratype **I** sternal plate of female paratype, ventral view **J** ovipositor of paratype, lateral view **K** hind femur of female paratype, ventral view.

**Male**: body medium-sized (Fig. 2A, B), head shorter than pronotum, and frons slightly oblique in profile. Frontal costa distinct and concave on the level of median ocellus (Fig. 2D). Vertical diameter of eyes 1.1 times that of transverse and equal to subocular furrows. Antennae filiform, with 23 segments, slightly longer than the head and pronotum combined; length of middle segment (12th segment) 1.2 times its width (Fig. 2C). Pronotum rough, nearly straight on anterior margin and slightly depressed on median posterior margin. Median carina distinct, slightly cut by 1st and 3rd transverse sulci; lateral carinae strongly distinct, lateral carinae parallel before 1st transverse sulci and later gradually expanding outward; prozona 2.1 times of metazona in length. Prosternal process conical and slightly sharp at apex. Mesosternal interspace at least 1.7 times wider than long (Fig. 2E). Metasternal lobes separated and distinct. Tegmina and wings absent. Upper carina of hind femur smooth. Hind femur 3.8 times longer than wide. Outer side of upper basal lobe of hind femur distinctly longer than lower one. Hind tibia with nine spines on inner and eight spines on outer side; external apical spine absent. Arolium between claws large, wide, round, reaching apex of claws. Posterior margin of 10th abdominal tergite without distinct furcula (Fig. 2E). Tympanal organ absent. Epiproct nearly triangular, longer than wide, bending inward at proximal part to form an obtuse angle terminally; epiproct with large denticles on the middle of both sides, denticles base wider than half of the distance between two denticles base, bluntly rounded at apex; epiproct with longitudinal groove basally, disappearing in the middle. Cerci process conical, sharp at apex, and not reaching tip of epiproct. Subgenital plate long, sharp-cornered, and bluntly rounded at apex (Fig. 2E). In phallic complex, length of apical valves of penis slightly exceeds that of cingulum valves; apodemes longer than basal valves of penis, terminating in sword-like ends. Llateral view of basal valves of penis reveals a reniform shape and distal apodemes (Fig. 4G–I). Bridge of epiproct straight, thin, and elongated; ancorae angular, extending forward, with non-sharp tips; anterior projections extending forward but not surpassing ancorae length; lateral plates straight and posterior projections extending outward. Lophi large and flaky, extending towards medial sides, protruding noticeably in overall plane. In dorsal view, they appear trapezoidal, with evident tumor protrusions along the edge (Fig. 4J–L). **Female**: body robust, larger than male in size (Fig. 2G, H). Vertical diameter of eyes 1.1 times that of the transverse diameter and 0.9 times that of subocular furrows. Mesosternal interspace at least 2.8 times wider than long (Fig. 2I). Ovipositor margin smooth, with a hook-like apex (Fig. 2J). Other characteristics resemble those of the male.

##### Coloration.

Body yellow or reddish-brown. Upper part of pronotum lobes darker in color than lower part. Two black spots on each of inner and outer sides of hind femur. Ventral face of hind femur black on outer side, red on basal 2/3 of inner and black on apical 1/3 of inner sides (Fig. 2K). Upper lateral genicular lobe black. Lower lateral genicular lobe dark yellow. Hind tibia purple above and dark brown below, with a lighter spot near the base of hind tibia. All tarsi yellowish, and sometimes 1st tarsomere purple.

##### Measurements.

Shown in Table 4.

**Table 4.** Measurement of *Kingdonellabiruensis* sp. nov.

Number of specimens	Male			Female		
	Body length	Pronotum length	Hind femur length	Body length	Pronotum length	Hind femur length
1	15.75	3.33	9.13	19.76	3.66	10.35
2	17.03	3.42	9.05	22.69	3.78	10.60
3	15.77	3.05	8.89	22.34	3.86	10.27
4	15.13	3.14	9.61	22.58	4.29	—
5	15.27	3.04	9.14	23.78	4.37	10.51
6	14.60	3.14	8.88	21.12	3.88	10.70
7	16.89	3.19	9.89	22.49	4.85	10.96
8	15.55	3.32	8.55	21.36	4.12	11.50
9	16.12	3.52	9.33	—	—	—
Min	14.60	3.04	8.55	19.76	3.66	10.27
Max	17.03	3.52	9.89	23.78	4.85	11.50
Median	15.75	3.19	9.13	22.46	4.00	10.60

#### ﻿ Kingdonella qinghaiensis

Taxon classificationAnimaliaOrthopteraAcrididae

Zheng, 1990

A4B0B9F5-5481-5D49-BEA1-D50B41B7C03F

Fig. 3

##### Examined material.

China • 4♂; Qinghai, Zhiduo; 33.76°N, 95.12°E, 19.VIII.2008; coll. Xinjiang Li, Jiantao Xiao, Yongchao Zhi; Catalogue number: #080819173 — #080819177. (first report of male)

##### Diagnosis.

The male *Kingdonellaqinghaiensis* resembles *K.parvula* Yin, 1984. The main differences are listed in Table 5.

**Table 5.** Comparison the male of *Kingdonellaqinghaiensis* Zheng, 1990 and *K.parvula* Yin, 1984.

Characters	*Kingdonellaqinghaiensis*	*K.parvula*
Width of mesosternal interspace	2.0 times than length	1.6 times than length
Outside of hind femur	Longitudinal dark spot present	Longitudinal dark spot absent
Hind tibia color	purple above and dark brown below	Yellowish-brown

##### Redescription.

**Figure 3.**
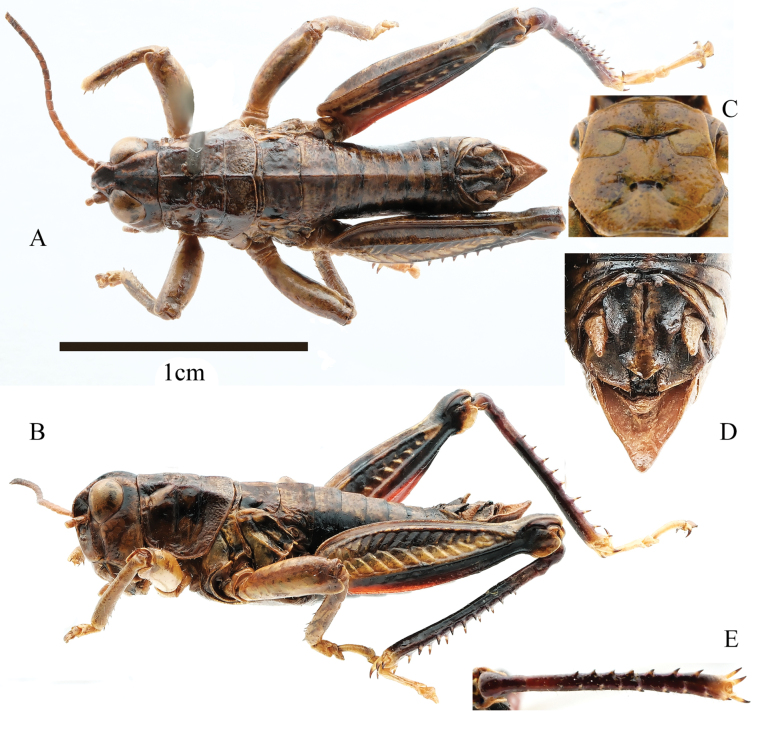
*Kingdonellaqinghaiensis* Zheng, 1990, male **A** dorsal view **B** lateral view **C** sternal plate, ventral view **D** terminal of abdomen, dorsal view **E** hind tibia, dorsal view.

**Figure 4.**
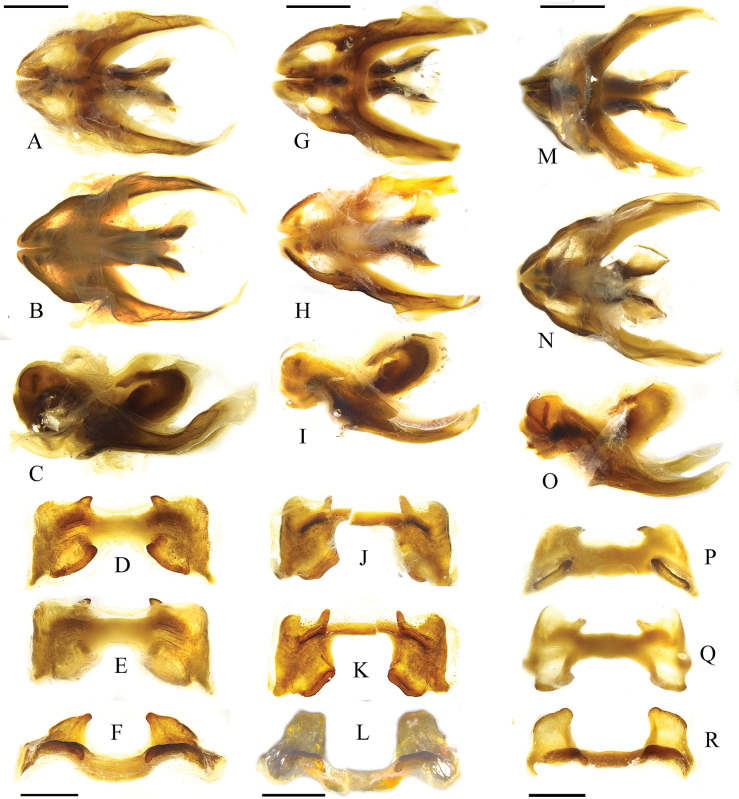
*Kingdonellagandensis* sp. nov. male **A–C** dorsal, ventral, and lateral views of phallic complex **D–F** dorsal, ventral, and axial views of epiphallus. *Kingdonellabaigasis* sp. nov. male **G–I** dorsal, ventral, and lateral views of phallic complex **J–L** dorsal, ventral, and axial views of epiphallus. *Kingdonellaqinghaiensis* male **M–O** dorsal, ventral, and lateral views of phallic complex **P–R** dorsal, ventral, and axial views of epiphallus.

**Male**: body small size (Fig. 3A, B). Head shorter than pronotum, with frons slightly oblique in profile. Frontal costa slightly shrunken in front of median ocellus. Eyes nearly elliptical, vertical diameter 1.2 times that of transverse diameter and subocular furrow length. Antennae filiform, 22–23 segments, longer than head and pronotum combined, length of a middle segment (12th segment) 1.5 times its width. Pronotum rough, slightly depressed medially on the anterior and posterior margins. Median carina distinct, lateral carinae nearly absent in metazona, lateral carinae cut by all transverse sulci and median carina cut by last transverse sulci; prozona 2.1 times longer than metazona. Prosternal process conical and blunt at apex. Width of mesothernal lobes 2.0 times the length (Fig. 3C). Width of mesothernal interspace 2.0 times length. Tegmina and wings absent. Upper median carina of hind femur smooth. Hind tibia with nine spines on inner and eight spines on outer sides; external apical spine absent. Arolium between claws large, nearly reaching apex. Posterior margin of 10th abdominal tergite with distinct furcula (Fig. 3D). Tympanal organ absent. Epiproct of male with larger denticles on middle of both sides; basally, denticles width nearly equal to half of length between denticles base, bluntly rounded at apex. Subgenital plate long and slightly sharp at apex (Fig. 3D). In phallic complex, length of the apical valves of penis slightly exceeds that of cingulum valves; apodemes longer than basal valves of penis, terminating in sword-like ends. The lateral view of the basal valves of the penis reveals a reniform shape and distal apodemes (Fig. 4M–O). Bridge of epiproct straight, wide, and short; ancorae angular, curving inward, with sharp tips; anterior projections not extending beyond length of ancorae; lateral plates incline outwards, and posterior projections extending outward. Lophi large and flaky, extending towards medial sides, protruding noticeably in overall plane. In dorsal view, inner length of the lophi longer than outer length, with evident tumor protrusions along edge (Fig. 4P–R).

##### Coloration.

Body reddish-brown. Area behind the eyes with a black band. Antennae brown, apex black. Lateral lobes of pronotum with a black spot in center; remaining brown. Outer side of hind femur with a long dark spot, inner side base red, remaining black. Median and apical 3/4 of hind femur dorsal face with dark spot; ventral face of hind femur red on inner and black on outer side. Hind tibia purple above and dark brown below (Fig. 3E). All tarsi yellow.

##### Measurements.

Shown in Table 6.

**Table 6.** Measurement of male *Kingdonellaqinghaiensis* Zheng, 1990.

Male			
Number of specimens	Body length	Pronotum length	Hind femur length
1	14.97	3.37	8.20
2	16.16	3.51	8.54
3	15.40	3.29	8.76
4	13.54	3.62	8.60
min	13.54	3.29	8.20
max	16.16	3.62	8.76
median	15.19	3.44	8.57

##### Distribution.

Zhiduo, Qinghai Province, China.

### ﻿Key to species of *Kingdonella* Uvarov, 1933 (Li and Xia 2006)

**Table:** 

1	In male, furcula at the end of 10th abdominal absent (Fig. 2F); epiproct with a transverse suture in the middle. Lateral carina of pronotum almost fully absent in both sexes, if distinct, epiproct of male with denticles on both ends of transverse suture	**2**
–	In male, furcula at the end of 10th abdominal tergite distinct (Fig. 1E), epiproct without transverse suture in the middle. Lateral carinae of pronotum at least partly distinct	**5**
2	Median carina of pronotum almost fully absent between 1st and 3rd transverse sulci in both sexes. Lateral carina of pronotum almost fully absent. Ventral face of hind femur dark yellow on inner side. Epiproct of male without denticles at both ends of transverse suture	***K.modesta***
–	Median and lateral carine in pronotum fully distinct (Fig. 2A). Ventral face of hind femur red on the inner side (Fig. 2K). Epiproct of male with denticles at both ends of transverse suture	**3**
3	Mesosternal interspace wider in male; minimum width 2.5 times as long as length. Epiproct of male with small denticles at both ends of transverse suture	***K.afurcula***
–	Mesosternal interspace same width in both sexes; minimum width 1.7 times as long as length. Epiproct of male with larger denticles at both ends of transverse suture	**4**
4	Subgenital plate of male sharp-cornered (Fig. 2F). Ovipositor smooth (Fig. 2J)	***K.biruensis* sp. nov.**
–	Subgenital plate of male bluntly rounded. Ovipositor with blunt spines in upper valve	***K.pienbaensis***
5	In males, epiproct without upward raised denticles in middle of both sides. Upper part of hind tibia in female greyish black	***K.saxicola***
–	In males, epiproct with upward raised denticles in middle of both sides (Fig. 1E). upper part of hind tibia of female not greyish black	**6**
6	Large and acute furcula at the end of 10th abdominal tergite in male. Epiproct of male with small denticles in middle of both sides. Ventral face of the hind femur in both sexes with only a small red spot at the base, remaining part black	**7**
–	Small and obtuse furcula at the end of 10th abdominal tergite in male. Epiproct of male with large denticles in middle of both sides. Ventral face of hind femur in both sexes, at least on basal half, bright red or dark red	**8**
7	Epiproct of male without longitudinal groove in middle of base	***K.hanburyi***
–	Epiproct of male with longitudinal groove in middle of base	***K.rivuna***
8	Eyes nearly circular in both sexes, vertical diameter nearly equal to transverse one in length	***K.pictipes***
–	Eyes elliptical in both sexes, vertical diameter 1.1–1.5 times as long as transverse one in length	**9**
9	Cerci longer, nearly reaching the tip of epiproct (Fig. 1E)	**10**
–	Cerci shorter, far from reaching the tip of epiproct	**11**
10	Hind tibia in both sexes bright red	***K.wardi***
–	Hind tibia in both sexes black (Fig. 1A)	***K.gandensis* sp. nov.**
11	Median carina of pronotum only cut by the 3rd transverse sulci in both sexes	**12**
–	Median carina cut by the three transverse sulci in both sexes	**14**
12	Hind tibia yellowish-brown	***K.parvula***
–	Hind tibia black or purple (Fig. 3E)	**13**
13	Median carina of pronotum fully distinct (Fig. 3A)	***K.qinghaiensis***
–	Median carina of pronotum indistinct between 1^st^ and 3^rd^ transverse sulci	***K.nigrotibia***
14	Ventral face of hind femur in both sexes bright red on inner side. Body small, in male less than 21 mm, in female less than 29 mm	**15**
–	Ventral face of hind femur dark red on inner side of basal 3/5 in female. Body large, length of body more than 30 mm in females	***K.maga***
15	Subgenital plate of male with conical spine projection at apex in male	**16**
–	Subgenital plate of male without conical spine projection at apex in male	**17**
16	Subgenital plate of male with short conical spine projection at apex. Posterior margin of end abdominal tergite with longe furcula. Frontal ridge almost parallel	***K.concia***
–	Subgenital plate of male with long conical spine projection at apex. Posterior margin of end abdominal tergite with shorter furcula. Frontal ridge slightly widened between antennae	***K.longiconica***
17	Inner sides of hind femora in both sexes black. Subgenital plate of male relatively wider at apex	***K.nigrofemora***
–	Inner sides of hind femora in both sexes yellowish-brown or yellow-green, with two indistinct dark spots on inner sides. Subgenital plate in male relatively more acute and slender	**18**
18	Epiproct of male with smaller denticles in middle of both sides; width at base less than 1/5 width of epiproct at denticles	***K.kozlovi***
–	Epiproct of male with larger denticles in middle of both sides; width at base less than 1/3 width of epiproct at denticles	***K.bicollina***

## Supplementary Material

XML Treatment for Kingdonella gandensis

XML Treatment for Kingdonella biruensis

XML Treatment for Kingdonella qinghaiensis

## ﻿Additional information

### Author ORCIDs

Jianyu Chen https://orcid.org/0000-0002-4993-3544

Yulong Zhang https://orcid.org/0009-0002-7983-515X

Xinjiang Li https://orcid.org/0000-0001-5204-0491

### Data availability

All of the data that support the findings of this study are available in the main text.

## References

[B1] Huang C (1981) Insects of Xizang, Orthoptera: Acrididae, Catantopinae, Pyrgomorphinae, Oedipodinae. 1. Science Press, Beijing, 82–83.

[B2] Li HC Xia KL (2006) Orthoptera, Acridoidea, Catantopidae. Fauna Sinica. Insecta 43: 431–451.

[B3] Li XJ Yin XC (2009) A taxonomic study of the subfamily Conophyminae (Orthoptera: Caelifera: Acridoidea) from Eurasia. Acta Entomologica Sinica 52(10): 1139–1144.

[B4] Liu L Wang Q Zhang Z He X Yu Y (2023) MATO: An updated tool for capturing and analyzing cytotaxonomic and morphological data. The Innovation Life 1(1): 1–7. 10.59717/j.xinn-life.2023.100010

[B5] Mishchenko (1952) Locusts and grasshoppers, Catantopinae. Fauna of the U.S.S.R. 4(2): 453–462.

[B6] Uvarov (1933) *Kingdonellawardi*, gen. et sp. n., a new grasshopper (Orthoptera, Acrididae) from the Assam Himalayas. Annals and Magazine of Natural History, London (Series 10) 11(64): 468–470. 10.1080/00222933308673677

[B7] Uvarov (1935) Three new grasshoppers from South-eastern Tibet (Orthoptera: Acrididae). Annals and Magazine of Natural History, London 10 15: 192–196. 10.1080/00222933508655036

[B8] Uvarov (1939) Some Acrididae from south-eastern Tibet. Zoological Journal of the Linnean Society 40(3): 561–574. 10.1111/j.1096-3642.1939.tb01693.x

[B9] Yin XC (1984) Grasshoppers and locusts from Qinghai-Xizang Plateau of China, Science Press, Beijing, 79–93.

[B10] Zheng Z (1980) New species of grasshoppers from China (Orthoptera: Acrididae). Acta Entomologica Sinica 23(2): 191–194.

[B11] Zheng Z (1990) Three new species of grasshoppers from Hengduanshan Range of China (Orthoptera: Acridoidea). Dong Wu Fen Lei Xue Bao 15(2): 197–200.

